# The Central Hospital Board Question

**Published:** 1897-01-30

**Authors:** 


### THE INSTITUTIONAL WORKSHOP.

### THE CENTRAL HOSPITAL BOARD QUESTION.

A special meeting of the Council of the Charity Organisation Society was held on Monday, 25th inst., at the Royal United Service Institute, Whitehall, S.W., under the chairmanship of the Earl of STAMFORD, "to consider the question of a Central Hospital Board for London, and to appoint a general committee to promote its establishment." The meeting was well attended.

The SECRETARY (Mr. C. S. Loch) read a telegram from H.R.H. the Princess Christian regretting that she was prevented from attending the meeting. Letters in favour of the idea of a Central Hospital Board for London were read from the Duke of Bedford, the Duke of Fife, the Earl of Cranbrook, the Earl of Meath, Sir James Paget, Sir Edward Sieveking, Sir James Crichton Browne, and Mr. J. Poland. Letters were also received from two of the gentlemen who were announced as speakers, viz., Mr. Pollock and Dr. Lauder Brunton, regretting their inability to attend. The latter thought that it would be well to give an outline of the position he would have taken had he been present to address the meeting. Since he had received Colonel Montefiore's draft scheme he had gone much more thoroughly into the *minutiæ* of the scheme, whereas previously he had only thought of its general bearings. While he still held the position that he did before, and regarded the formation of a Central Hospital Board for London, with due representation of the medical and lay elements upon it, as very desirable, yet he felt sure that no scheme would succeed which did not meet with the hearty approbation of those who were devoting their time and energies to the alleviation or cure of disease, either directly by the treatment of patients, or indirectly by hospital management. The scheme, as at present drafted by Colonel Montefiore, did not, so far as he could learn, meet with sufficient approval from either of these classes to succeed. He, therefore, suggested that, instead of pressing for the adoption of the draft scheme, it would be best to be content with (1) reaffirming the decision of the previous meeting that a Central Hospital Board for London is desirable; (2) that in it the medical and lay element should both be represented; and (3) that the governing bodies of the various charities in London should be approached with the object of learning their views regarding the constitution of such a board, and their willingness to co-operate in its formation. He felt strongly that the draft submitted was not likely to prove satisfactory, and had he been able to be present at the meeting he would have felt himself obliged to move an amendment to that effect.

The CHAIRMAN, after briefly tracing what had been done in the past with reference to this question, said that it was felt that the time had arrived to go a step further forward in the scheme. It was nearly a year ago since he had presided at a meeting at which the principle was affirmed that a Central Hospital Board for London was desirable. The present meeting was called in the hope that the time might be ripe to go further towards realising that principle, and to appoint a committee to promote it. In some of the letters read there seemed to be a slight misconception as to the object of that meeting. That meeting was not to form a Central Hospital Board, but only to pave the way towards such a board being appointed. After explaining the principal points of the scheme proposed by the Charity Organisation Society, he concluded with the remark that there might be other schemes or designs in existence at the present moment with the same end, but if their plan, after all others had been discussed and weighed, was the better one, then it would hold its own ground; but if any other plan was better than theirs then they would be quite prepared to give way to it.

Sir WILLIAM BROADBENT, Bart., proposed the following resolution:—

"That this meeting affirms the resolution adopted on the 13th April last, approving of the establishment of a Central Hospital Board for London on a representative basis and accepts the draft submitted to it as indicating a plan that, subject to further consideration and modification, is likely to prove satisfactory; and that the following be a general committee to consider, promote, and from time to time report upon the draft scheme, with power to add to their number and to appoint an executive committee."

[Here follows a list of about 150 names.]

He need hardly say that nothing in the proceedings of that meeting could be taken as hostile to hospitals. After briefly pointing out the abuses which had arisen in regard to hospital work, especially in the out-patient departments—a matter which might not concern the public so closely, but which was one of great interest to the medical profession—he proceeded to discuss the causes which had contributed to this state of things. One of the main causes was the isolation of the great hospitals from each other. Owing to the competition which had sprung up among them there had been no intercommunication, no comparison of work or methods among the great hospitals, and thus had arisen diversities to begin with and increasing divergencies which had left gaps, and those gaps had sometimes been filled by small institutions which had, many of them, done great harm. (Hear, hear.) Since one of the great causes of the difficulties they had now to face was due to the isolation and competition of hospitals, one means of remedy must be co-ordination. There must be nothing to hinder and restrict the energy of different hospitals, no idea of extinguishing the rivalry between them; but the rivalry must be in the direction of the good of the patients, and not merely in that of increasing their numbers and being able to put forward specious claims to the support of the public founded on numbers, and not founded on efficiency or on the value of the work accomplished. (Applause.) He would like to see a local co-ordination—that each large hospital and its medical school should be a centre of usefulness, with which should be affiliated the provident dispensaries which represented the better and more highly principled among the working classes who were willing to pay for their medical advice. He would say the same even with regard to friendly societies. They actually helped the hospitals, and they ought to get something more in return than a certain number of out-patient letters. With regard to the question of the formation of a central board, he confessed himself entirely out of his depth. The functions of such a board were clear enough, and he thought they had been set forth clearly in the scheme which had been laid before them. It was no compulsion—simply obtaining information from all those institutions, and, as far as possible, with that information, giving encouragement in the form of money grants for efficiency—efficiency not merely by statistics and accounts, but by the reports of visitors. He did not suggest any modifications of the scheme, as this was not the time to do that; but he assured his hearers that nothing was to be done against the general opinion of those who were working in the hospitals. The first object of the general committee would be to obtain the co-operation of the hospitals. The speaker concluded with a reference to the possibility of there being a gigantic scheme for the benefit of hospitals, inaugurated by the Prince of Wales in commemoration of the Queen's reign.

Mr. VICTOR HORSLEY, F.R.S., formally seconded the resolution.

Mr. MYERS moved the following amendment:—

"That the Charity Organisation Society draw up recommendations to the boards of the London hospitals, &c., to enable those boards as much as possible to compare their relative expenditure and to profit when expedient thereby; and also to enable the Charity Organisation Society to draw up accurate annual reports and statements of accounts of those charities. (2) That this society issue such advice as they may deem expedient for the better regulation of the out-patient department of the various hospitals and dispensaries."

This was seconded.

Mr. VILLARS asked if this scheme had been submitted to the governing bodies of the twelve large hospitals in London in order that they might express an opinion on it in a united form.

The SECRETARY said that the scheme had been circulated so far chiefly among the medical profession, as in the first instance it was desired to obtain their opinion about it.

Rev. Dr. WACE, chairman of King's College Hospital, regretted that that motion had been brought before the hospitals on such short notice. It had only reached his hospital on the previous Tuesday. As the weekly meeting of his board was held on Wednesday, it was impossible for them to form a definite opinion on it. Although the resolution did not ask them definitely to approve of the scheme, yet it did ask the meeting to take it as a satisfactory basis. He confessed that as the scheme stood, although he would be sorry absolutely to oppose it, yet he felt very great hesitation in expressing anything which could be taken to mean that it was accepted as generally satisfactory. (Hear, hear.) His reason for that was that he could not resist the impression that it was a scheme for the constitution of a governing body; and to ask hospitals like his to put themselves under a governing body was a very serious thing indeed. It became all the more serious when one looked at the constitution of that governing body. Any co-operation of the London hospitals must at the outset at any rate be of the nature of a consultative co-operation.

The amendment having been put was lost, only ten voting for it.

Mr. C. B. KEETLEY then moved the following amendment:—

"That this meeting affirms the resolution adopted on the 13th of April last approving of the establishment of a Central Hospital Board for London on a representative basis, and suggests that all further steps taken in the establishment of such board should be taken by a committee of representatives duly elected by the individual authorities of the hospitals. That Lord Stamford, Sir William Broadbent, and Mr. Loch be requested to adopt the necessary initiative steps in this matter."

Dr. MORGAN DOCKERELL, criticising the composition of the general committee, said that the result would be that but few of the committee would turn up at the meetings, and the work would be carried on by the representatives of the Charity Organisation Society.

Mr. TAYLOR thought the amendment was a waste of time. The resolution would be sent to the hospitals, and on meeting again it would be found that with one or two exceptions the hospitals had replied that they preferred to manage their own affairs. Hospital boards did not want to abolish out-patients, as out-patients meant money. They argued if $x$ out-patients bring in $y$ money, then 2 $x$ will bring in 2 $y$, therefore let us have 2 $x$ out-patients. (Laughter.)

The amendment on being put was lost, twenty-three voting for it.

Mr. TIMOTHY HOLMES did not think so important a speech as that of Dr. Wace's should be neglected. He therefore thought that someone in authority should assure Dr. Wace that every hospital in London would have the most ample opportunity of consulting and being consulted about every detail of the scheme before any final resolution was taken upon it. (Cries of "No, no," and "Yes, yes.") He was surprised to hear some gentlemen say "No, no," to his request, as he had thought he was almost trespassing on the time of the meeting in stating so elementary a proposition. (Laughter.)

The SECRETARY said that there was no intention whatever of settling anything in regard to hospitals without full reference to them.

The resolution was put to the meeting, and carried, 121 voting for it and 12 against.

The meeting, which lasted nearly two hours, concluded with a vote of thanks to the chairman.

